# Detraining among Athletes—Is Withdrawal of Adaptive Cardiovascular Changes a Hint for the Differential Diagnosis of Physically Active People?

**DOI:** 10.3390/jcm13082343

**Published:** 2024-04-18

**Authors:** Kinga Zujko-Kowalska, Karol Adam Kamiński, Łukasz Małek

**Affiliations:** 1 Department of Population Medicine and Lifestyle Diseases Prevention, Medical University of Bialystok, Waszyngtona 15B, 15-269 Białystok, Poland; karol.kaminski@umb.edu.pl; 2 Department of Cardiology and Internal Medicine with Cardiac Intensive Care Unit, Medical University of Białystok, M. Skłodowskiej-Curie 24a, 15-276 Białystok, Poland; 3 Faculty of Rehabilitation, University of Physical Education, Marymoncka 34, 00-968 Warsaw, Poland; lukasz.malek@awf.edu.pl

**Keywords:** athletes, detraining, adaptive changes, cardiomyopathy, sports cardiology, differentiation

## Abstract

An athlete’s training aims to achieve the highest possible sports results by improving physical dispositions which lead to cardiac adaptive changes. The annual training cycle is divided into periods. The preparatory period begins with gradually increasing training intensity and volume until the competitive period occurs, when the athlete’s maximum performance is expected. Finally, the athlete enters a phase of loss of fitness, which is called detraining. Detraining is a time of resting both physically and mentally from the training regime and usually lasts about 4 weeks for endurance athletes. We collected data from much research on athletes’ detraining. According to these data, the earliest change after detraining seems to be a decrease in left ventricular wall thickness and left ventricular mass, followed by decreased performance parameters, diastolic diameter of the left ventricle and size of the left atrium. A reversal of adaptive changes affects the left heart chamber first, then the right atrium and, finally, the right ventricle. Training reduction is often proposed as a method of differentiating an athlete’s heart from cardiomyopathies. The aim of this study is to consider the diagnostic value of detraining in differentiating athletes’ hearts from cardiomyopathies. We suggest that detraining cannot be conclusive in differentiating the disease from adaptive changes. Although a withdrawal of the characteristic morphological, functional and electrocardiographic changes occurs in healthy athletes during detraining, it can also concern individuals with cardiomyopathies due to the lower expression of abnormal features after decreased training loads. Therefore, a quick diagnosis and individual assessments using imaging and genetic tests are essential to recommend a proper type of activity.

## 1. Introduction

An athlete’s training aims to achieve the highest possible sports results by improving their physical and mental dispositions. The total training load is distributed into periods in a macrocycle lasting from half a year to even more than a year. This training time structure consists of mesocycles, microcycles and finally, single training units. The first stage of the annual training cycle is the preparatory period, in which the training intensity gradually increases. Awhile before the start of the first competition, the competitive period begins, and then the athlete’s performance should be at the highest level. Strenuous physical effort leads to disruption of homeostasis, and systematic training causes adaptive changes which apply to the entire organism, especially the cardiovascular (CV) system and skeletal muscles. These changes emerge during a phase of building sports form. The response of an athlete’s organism to long-term training is different in different types of training, but factors such as gender, age, training level, duration of training and detraining also influence the clinical picture. The division of disciplines into skill, strength, mixed and endurance has been proposed according to the dominant component of the sport’s category [1]. Endurance sports with the dominance of dynamic components contribute the most to adaptive CV changes compared to other types of activity [2]. After the starting period, the athlete enters a phase of loss of form, which is called detraining. Detraining is a time of resting both physically and mentally from the training regime and usually lasts about 4 weeks for endurance athletes. It normally does not involve a complete withdrawal from exercises, but it is based on a reduction in volume and intensity, with a shift towards activities other than the target sport. The undeniable advantage of this period is the prevention of the injuries to which an overtrained body is exposed, and it allows the release of all the mental and physical stress accumulated during the starting season. In general, physical fitness regress is observed after the detraining period. Along with a decline in physical performance, a reduction in some adaptive changes in the cardiovascular system may also be observed. It is considered that detraining is a good tool to distinguish sports-related adaptive changes from pathology. The aim of this study is to show the uncertain diagnostic value of detraining in differentiating the heart of a healthy athlete from cardiovascular diseases.

## 2. The Prolonged Impact of Endurance Training on the CV System

**Figure 1:**
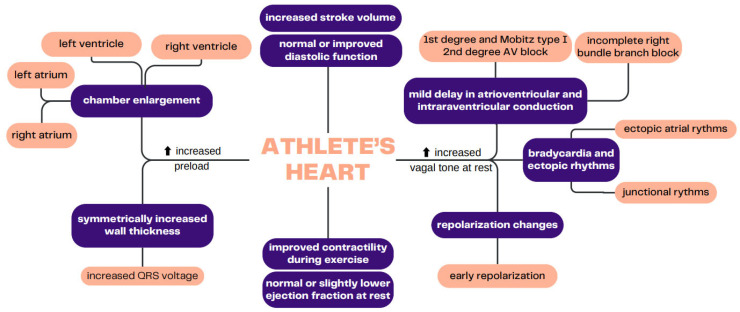
Main components of an athlete’s heart phenotype that are visible in electrocardiogram and imaging studies.

Endurance training is characterized by an increased preload due to venous return, which is affected by skeletal muscle and the respiratory pump. In the long term, increased volume overload during bouts of intensive exercise causes eccentric remodeling of the myocardium. It results in balanced enlargement of both ventricles and atria with slight, symmetrical or no apparent increase in the wall thickness (Figure 1). These changes, according to the Frank–Starling law, permit athletes to achieve higher cardiac output during exercises, as an increased end-diastolic volume of the ventricles leads to increased stroke volume. Another common occurrence is also a slightly lower ejection fraction at rest and improved filling of the heart with blood during diastole [3]. Altogether, the larger volume of the cardiac chambers, higher volume reserve and supranormal diastolic function in athletes result in a higher stroke volume than in inactive people. The volume load during endurance exercises particularly affects the right ventricle because it has less ability to reduce the increased afterload than the left ventricle; therefore, the right ventricle is more vulnerable to damage. The commonly known electrographic changes that can be observed therefore represent the main features of this balanced cardiac enlargement but also demonstrate the predominance of the influence of the parasympathetic system on the heart. These most often include sinus bradycardia or ectopic atrial/junctional rhythms, mild delay in atrioventricular and/or intraventricular conduction (incomplete right bundle branch block) and early repolarization.

Strength training has a different effect on the human body than endurance training. The greater afterload results in an increase in arterial pressure and more concentric remodeling of the heart. Systematic training also leads to hypertrophy of the working skeletal muscles, a change in the type of muscle fibers and an increase in dynamic strength [4].

## 3. Effects of Detraining in Endurance Sports

### 3.1. Cardiac Morphology

Detraining is a time of reducing physical activity below the previous threshold. It is part of the training cycle and lasts about 2–4 weeks for endurance athletes. Detraining may consist of a partial reduction in volume, training intensity or both, a change in the type of sport or a complete discontinuation of exercise. Lack of training may also be the result of an injury or the end of a career in professional sport. Deconditioning is the reduction in adaptation to training after the loss of the training stimulus. Studies on young endurance athletes with cardiac hypertrophy and excluded hypertrophic and dilated cardiomyopathy showed that left ventricular diastolic dimension (LVDd), left ventricular wall thickness and left ventricular mass can significantly decrease after detraining lasting only a few weeks. LV mass and wall thickness have a greater tendency to decrease in comparison to left ventricle dimensions [5,6]. In addition, some people, especially competitive athletes, may experience incomplete recovery of adaptive changes due to a prior higher training load and more pronounced adaptive changes. A study based on long-term observations of 1 year to 13 years of detraining showed that 80% of athletes had reduced LV dimensions caused by training cessation. Typically, the diameter reduction was in the range of 2 to 5 mm [5].

Nonetheless, the LVDd showed a tendency towards incomplete regression. Even after long detraining, this parameter was still at the upper limit of normal (>55 mm) in most professional athletes and even exceeded 60 mm in 20% of them. This incomplete withdrawal of ventricular enlargement was associated with maintaining recreational activity during detraining and increased body mass [5]. LVDd were already significantly reduced by an average of 9–10% after 3 weeks [6,7]. Left ventricular end-diastolic volume (LVEDV) also decreased after 3 weeks, with no changes within 1 week, as was observed in case of left ventricular end-systolic volume (LVESV), which may explain the slight increase in the ejection fraction during this time [8].

The significant reduction in LV wall thickness already begins to reveal itself after 1 week [9]. Studies based on small groups of endurance athletes with reduction in LV wall thickness after 3 weeks revealed that the decline of this parameter progressed in subsequent follow-ups [6,7]. Decreases of 12.5% and 25% in the thickness of the posterior LV wall were demonstrated after 3 and 6 weeks [6]. A reduction of 15–33% in ventricular septal thickness was also observed during the period of 6–34 weeks after detraining [9]. In a larger study based on a long follow-up period of 1–13 years, elite athletes had an average reduction of 15% in their ventricular septal walls [5]. In recreational marathon runners, a decrease of 8% in the LV wall was observed within 4 weeks [10]. Differences in percentages may result not only from the number of participants but also from the lower tendency of cardiac changes in amateurs compared to the elite [11]. Therefore, detraining leads to a decrease in LV wall thickness, usually of ≥2 mm, and in some athletes, this parameter can be reduced by 0–1 mm or even be over 5 mm [5,9,10]. At longer times after withdrawal from competitive sport, no wall hypertrophy above 12 mm was detected in any of the athletes [5].

Left ventricular mass is directly positively correlated to LV size and thickness. The beginning of left ventricular mass regression has already been observed after 1 week [8,11]. Detraining lasting 3 weeks resulted in a decrease in left ventricular mass of almost 20% [10]. In a long-term study on elite athletes, a 28% reduction in this parameter was achieved, although in almost half of the individuals, it was still elevated even after a few years [5]. The reduction in left ventricular mass during 3–4 weeks in both elite and recreational, endurance and mixed-sports athletes, showed no further changes in the following weeks [6,9,10,12]. A study on two groups of cyclists of different ages showed that the changes associated with 2 months of inactivity were similar regardless of age, but young athletes had a greater tendency towards reduction in left ventricular wall thickness and older athletes towards reduction in the LV mass and diameter [13]. A good summary of structural changes in the heart after various periods of detraining is included in a table in review [14].

Fewer data are available about changes in both atria and the right ventricle after detraining. A decrease in atrial dimensions can be observed after 3 weeks [10]. However, dimensions not exceeding the reference values were obtained only after 6 months [15]. The right heart, which is more susceptible to increased training load, also appears to respond later to detraining. The right atrium area decreased in marathon runners within 4 weeks of inactivity. A reduction in the right ventricle chamber size (end-diastolic dimension or length) was observed after 8 weeks, with no changes in RV ejection fraction [10].

### 3.2. Electrocardiographic Parameters

Electrocardiographic parameters associated with hypertrophy may recede simultaneously with echocardiography parameters. R wave voltage decreases in the lead V5 in ECGs, and as a consequence, the lack of Sokolow-Lyon criteria can be visible after 4 weeks of detraining in athletes [12]. Another study showed a decreased S wave in the lead V1 and V2 in 3 weeks, with no changes in the R wave [8]. In a long-term study, decreased PR and QTc and an increased heart rate (HR) at rest were also observed [5]. A loss of bradycardia was presented in a study based on rats after 2 weeks of detraining [16].

### 3.3. Functional Parameters

**Figure 2:**
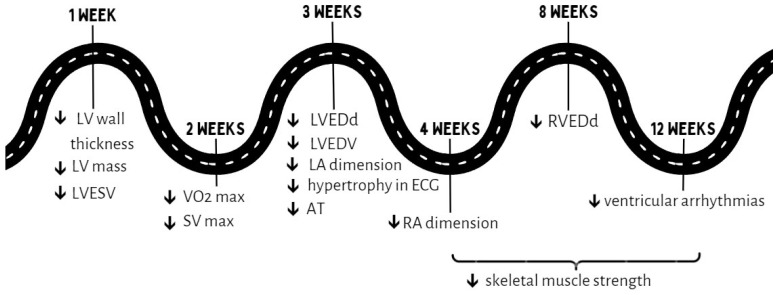
The earliest time of withdrawal of adaptive changes according to available research on healthy athletes. LV—left ventricle, LVESV—left ventricular end-systolic volume, LVEDV—left ventricular end-diastolic volume, VO_2_—oxygen uptake, SV—stroke volume, AT—anaerobic threshold, LA—left atrium, RA—right atrium, LVEDd—left ventricular end-diastolic diameter, RVEDd—right ventricular end-diastolic diameter.

The physiological effects of detraining seem to appear earlier than most morphological changes [17]. Detraining for 3 weeks by endurance athletes may result in a decrease in maximum oxygen uptake (VO_2_ max) [6,7] and stroke volume (SV) [6] but generally has no significant impact on the ejection fraction and diastolic function [5,7]. Interestingly, there is some information about an increase in the ejection fraction after detraining [8], which may be explained by a slightly lower ejection fraction in athletes at rest. Studies on young male endurance athletes suggested a significant decrease in physical performance even in a shorter period of 2–4 weeks, as detraining resulted in a 6–7.5% decrease in VO_2_ max [18,19,20,21], 12% decrease in SV max [18] and 14% decrease in the anaerobic threshold (AT) [20], with a significant correlation between these parameters and VO_2_ max [18,19,20]. Further observation after 8–9 weeks showed a VO_2_ max that was reduced by 11–16% [20,21] and an AT that was almost 20% lower [20]. However, both the absolute VO_2_ max and the AT did not return to the levels that were measured before training, even after 8–9 weeks [20,21]. No further decline in VO_2_ max was observed after inactivity of longer than 3 months [22]. The values of SV finally returned to the level of the control group, but muscle capillarization and oxidative enzyme activity still remained increased [21]. In long-term detraining, a significantly greater tendency towards reduction in VO_2_ max was observed in young athletes <20 years old and individuals with higher VO_2_ max values before training cessation than in those of older ages and with lower fitness levels. After a short period of inactivity (<30 days), the decrease in VO_2_ max was independent of age and previous fitness level [22]. Partial reduction in physical activity has a smaller impact on the withdrawal of adaptive changes than complete inactivity but only in long-term detraining. Not completely ceasing training can allow athletes to maintain a certain level of physical performance when they have to limit physical activity for more than one month [22]. However, the type of physical activity during incomplete detraining had an important impact on athletes’ performance. A large influence on preserving fitness levels, even after 2 weeks, has been demonstrated for high-intensity physical effort, without the necessity to maintain training volume [22,23,24]. We propose a scheme with the earliest time of withdrawal of adaptive changes according to available research on healthy athletes (Figure 2).

## 4. Effects of Detraining in Resistance Sports

Strength achieved with resistance training lasts longer after detraining than the effects of endurance sports. However, available research has been based on short training periods before deconditioning. Moreover, there have been no studies indicating the duration of detraining for this effect to occur. Some studies have shown that stopping strength training for 12 weeks does not diminish muscle strength and strength endurance or causes only a slight decrease [25,26] despite a significant tendency to lose muscle mass [27]. However, even after this period, dynamic muscle strength still remains greater than before the start of training. It may occur due to a decrease in the involvement of motor units and changes in the size of the type II fibers [26,28,29]. Additionally, changes in types of fibers were observed as a result of detraining. Since resistance training converts type IIx muscle fibers to IIa, the detraining period changes the ratio of these fibers in the opposite way [28]. During shorter periods of detraining, a reduction in muscle glycogen levels may be also responsible for muscle atrophy. After 2 weeks of detraining, a significant decrease in the surface electromyogram activity of some muscles was observed [29]. An inactivity period leads to increased levels of growth hormone (even more than 50%), testosterone and the testosterone-to-cortisol ratio, with a significant decrease in cortisol [29,30] and enzyme creatine kinase levels. These changes may correspond to an increase in anabolic processes, however, without the effect of muscle hypertrophy due to the lack of training stimulus [29]. In young women, the cessation of training causes a decrease in bone mineral density to baseline values [31]. Gender has no impact on the effects of 12 and 36 weeks of detraining, but older age is more predisposed to the loss of muscle strength [25]. Partial loss of muscle power in women > 50 years old can already be revealed after 4 weeks of inactivity [32]. However, in older groups of both men and women, the effects of strength training were not completely reversed even after 12 weeks [33,34].

## 5. Cardiovascular Diseases

Appropriately selected physical activity is recommended for people with cardiovascular diseases. Some of the many benefits of physical activity include lower blood pressure, improvement of lipid profile, mass reduction, increased exercise tolerance and decreased risk of cardiac infarction or heart failure [35,36,37]. Physical exercise is a stimulus for oxidative stress and therefore leads to greater activation of antioxidant enzymes [38]. Unfortunately, after cessation of training, most of the positive effects on redox status disappear after 3 months of detraining [36]. In hypertensive patients with higher cardiovascular risk, lipid profiles and cardiorespiratory fitness, which were previously improved by exercise, returned to baseline values after 7 weeks of inactivity [39]. However, the blood pressure-lowering effect of training seems to last for several months. A study on patients with atrial fibrillation who underwent 3 months of detraining after cardiac rehabilitation showed that this period of inactivity had an impact on decreased exercise tolerance and quality of life [35].

Despite the health benefits of physical activity and the reduced risk of death, very high-intensity sports are associated with the risk of revealing undesirable features. The correlation between physical activity and its benefits is described as U-shaped [40]. If the volume and intensity of exercise exceeds the regenerative capacity, further exercise has a negative impact on the cardiovascular system. Especially when undiagnosed heart disease is combined with hard training, it can cause worsening of the symptoms of the undiagnosed disease and lead to sudden cardiac death (SCD). Among athletes, the challenge is to differentiate an athlete’s heart from cardiovascular diseases, which is sometimes difficult due to some similar/overlapping features. Among young athletes, the most common causes of SCD are cardiomyopathies (especially hypertrophic cardiomyopathy, but also arrhythmogenic right ventricular and dilated, restrictive compaction cardiomyopathy), coronary artery anomalies, ion channel diseases or infective causes [41]. One method of identifying the pathology of athletes’ hearts based on detraining assumes that adaptive changes in healthy athletes should reverse, but the cessation of training should not significantly affect cardiac morphology, function and ECG changes in cardiomyopathies [42]. However, the phenotypic expression of disease can intensify due to the influence of intensive exercise. Moreover, the features of cardiomyopathy may also be reduced after cessation of training, which questions the utility of detraining as part of the diagnostic process.

Among young people, the most common direct cause of SCD is arrhythmogenic sudden death [43]. Among athletes with genotypic predisposal towards arrhythmogenic right ventricular cardiomyopathy (ARVC), high-intensity endurance training is associated with an earlier onset of symptoms and a higher incidence of ventricular arrhythmias compared to people who perform less exercise [44]. Some research suggests that withdrawal of premature ventricular contractions after detraining is a characteristic feature of an athlete’s heart [45]. However, a reduction in the frequency and complexity of ventricular arrhythmias, both in healthy athletes and those with the presence of structural heart disease, was already obtained after 12–24 weeks of inactivity [46]. In ARVC, training cessation results in a 60% reduction in ventricular arrhythmia events and premature ventricular contractions (PVCs) after several years [47]. Greater training restriction is associated with fewer ventricular arrhythmias [48]. After a 9-year observation of athletes with hypertrophic cardiomyopathy (HCM), no differences were revealed in the frequency of ventricular arrhythmias in the training and non-training groups [41]. Training cessation over 6 months also caused a reduction in the thickness of the left ventricular walls (posterior wall, septum and apex) and an improvement in longitudinal strain deformation in an athlete with a high probability of HCM [49]. After 18 months of detraining, an athlete with a genetic diagnosis of HCM achieved both a reduction in hypertrophy and complete ECG normalization [50]. Unusual electrographic features of cardiac adaptation, such as inversion of the T wave in the posterior and lateral leads, can be withdrawn within 2 months [51,52]. This suggests that detraining cannot be conclusive in differentiating disease from adaptive changes, especially with the availability of various imaging methods and genetic tests.

According to guidelines [1], most cardiac diseases are not an indication to completely stop training and it is recommended to regularly engage in physical activity which is appropriate to the health condition after risk assessment. Cardiac rehabilitation and individually tailored physical effort can improve the physical capacity [53,54], VO_2_ peak and left ventricular ejection fraction of patients with cardiomyopathies [54]. Therefore, a quick diagnosis of the disease is essential to recommend a proper type of activity. An individual assessment is essential in allowing athletes to participate in sports with various intensities.

## 6. Summary

Detraining is an important part of an athlete’s training cycle, not only for physical reasons but also for the athlete’s mental rest. However, in light of the recent data, the usefulness of observation of morphological changes during detraining in diagnosing athletes with potential CV disease is questionable. Although people with cardiomyopathies should adjust the intensity and type of sport according to recommendations to reduce the load on the affected cardiac muscle, reversing adaptation during this period is not always desirable for healthy athletes due to deterioration of performance. After short periods of detraining, athletes quickly return to their previous form [55]; therefore, 2–4 days of detraining are recommended in the training cycle. Long-term deconditioning has a more negative impact on athletes’ physical performance [23]. Moreover, athletes in the period of detraining are recommended not to completely stop physical activity, but to reduce it or change the type of sport. The earliest change after detraining seems to be a decrease in left ventricular wall thickness and left ventricular mass, followed by decreased performance parameters, diastolic diameter of the left ventricle and size of the left atrium. Reversal of adaptive changes first affects the left heart chamber, then the right atrium and, finally, the right ventricle.

A withdrawal of the characteristic features of morphological, functional and electrocardiographic changes during detraining not only affects healthy athletes, but it can also concern individuals with cardiomyopathies due to the lower expression of abnormal features after a decreased training load. Despite studies showing a lack of influence of deconditioning on the withdrawal of changes associated with cardiomyopathies, there are case reports of a reduction in arrhythmia in ARVC [46,47,48] and a decrease in pathological wall thickness after restriction of intensive physical activity among patients with HCM [41,49,50]. Therefore, it seems that reduction in intense physical activity cannot be a good tool for differentiating an athlete’s heart from cardiovascular diseases, and excluding HCM based only on reducing the wall thickness after detraining carries the risk of a false negative result. Moreover, the restriction of sports is a mental burden for the athlete, without the certainty of a diagnosis. More research is needed at this point to compare the impact of cessation of high-intensity endurance physical exertion on the reverse remodeling of cardiomyopathies, as pathologic phenotypes, contrary to what was previously believed, may also regress after exercise stimulus withdrawal. Any “gray zone” changes or unusual findings for athletes always require further diagnostics, but in our opinion, detraining as a diagnostic method should not be considered as a first line anymore. In the case of athletes suspected of having cardiomyopathy, other features should be taken into account in the first place, including their thorough family history, clinical symptoms, the occurrence of arrhythmias at rest and during exercise, laboratory findings, atypical electrocardiographic changes, atypical echocardiographic findings, cardiac magnetic resonance changes including signs of diffused or localized myocardial fibrosis and genetic testing.

## 7. Future Directions

Currently, there are gaps in the research on how detraining affects the cardiovascular system in people with heart diseases. Not only in healthy athletes, but also in the case of cardiomyopathy, features and symptoms may regress after withdrawal from training. There are mainly single case reports that reveal that detraining does not differentiate well between cardiac disease and adaptive changes. There is no study that compares both groups over similar periods of time. Moreover, the available research does not show changes during entire annual training cycles of athletes but only the effects of imposed detraining or training for a specific period of time. There is also still a deficit of research on women.

## Data Availability

Not applicable.

## References

[1] Pelliccia A., Sharma S., Gati S., Bäck M., Börjesson M., Caselli S., Collet J.P., Corrado D., Drezner J.A., ESC Scientific Document Group (2021). 2020 ESC Guidelines on sports cardiology and exercise in patients with cardiovascular disease. Eur. Heart J..

[2] Utomi V., Oxborough D., Whyte G.P., Somauroo J., Sharma S., Shave R., Atkinson G., George K. (2013). Systematic review and meta-analysis of training mode, imaging modality and body size influences on the morphology and function of the male athlete’s heart. Heart.

[3] Boraita A., Sánchez-Testal M.V., Diaz-Gonzalez L., Heras M.E., Alcocer-Ayuga M., de la Rosa A., Rabadán M., Abdul-Jalbar B., Pérez de Isla L., Santos-Lozano A. (2019). Apparent ventricular dysfunction in elite young athletes: Another form of cardiac adaptation of the athlete’s heart. J. Am. Soc. Echocardiogr..

[4] Encarnação I.G.A., Viana R.B., Soares S.R.S., Freitas E.D.S., de Lira C.A.B., Ferreira-Junior J.B. (2022). Effects of detraining on muscle strength and hypertrophy induced by resistance training: A systematic review. Muscles.

[5] Pelliccia A., Maron B.J., De Luca R., Di Paolo F.M., Spataro A., Culasso F. (2002). Remodeling of left ventricular hypertrophy in elite athletes after long-term deconditioning. Circulation.

[6] Martin W.H., Coyle E.F., Bloomfield S.A., Ehsani A.A. (1986). Effects of physical deconditioning after intense endurance training on left ventricular dimensions and stroke volume. J. Am. Coll. Cardiol..

[7] Ehsani A.A., Hagberg J.M., Hickson R.C. (1978). Rapid changes in left ventricular dimensions and mass in response to physical conditioning and deconditioning. Am. J. Cardiol..

[8] Petretta M., Cavallaro V., Bianchi V., La Mura G., Conforti G., Breglio R., Valva G., Morgano G., Bonaduce D. (1991). Modificazionicardiacheindotte dal decondizionamentonell’atleta: Studio ecocardiografico ed elettrocardiografico [Cardiac changes induced by deconditioning in athletes: An echocardiographic and electrocardiographic study]. G. Ital. Cardiol..

[9] Maron B.J., Pelliccia A., Spataro A., Granata M. (1993). Reduction in left ventricular wall thickness after deconditioning in highly trained Olympic athletes. Br. Heart J..

[10] Pedlar C.R., Brown M.G., Shave R.E., Otto J.M., Drane A., Michaud-Finch J., Contursi M., Wasfy M.M., Hutter A., Picard M.H. (2018). Cardiovascular response to prescribed detraining among recreational athletes. J. Appl. Physiol..

[11] Elliott P.M., Anastasakis A., Borger M.A., Borggrefe M., Cecchi F., Charron P., Hagege A.A., Lafont A., Limongelli G., Mahrholdt H. (2014). 2014 ESC Guidelines on diagnosis and management of hypertrophic cardiomyopathy: The Task Force for the Diagnosis and Management of Hypertrophic Cardiomyopathy of the European Society of Cardiology (ESC). Eur. Heart J..

[12] Swoboda P.P., Garg P., Levelt E., Broadbent D.A., Zolfaghari-Nia A., Foley A.J.R., Fent G.J., Chew P.G., Brown L.A., Saunderson C.E. (2019). Regression of left ventricular mass in athletes undergoing complete detraining is mediated by decrease in intracellular but not extracellular compartments. Circ. Cardiovasc. Imaging.

[13] Giada F., Bertaglia E., De Piccoli B., Franceschi M., Sartori F., Raviele A., Pascotto P. (1998). Cardiovascular adaptations to endurance training and detraining in young and older athletes. Int. J. Cardiol..

[14] Petek B.J., Groezinger E.Y., Pedlar C.R., Baggish A.L. (2022). Cardiac effects of detraining in athletes: A narrative review. Ann. Phys. Rehabil. Med..

[15] Weiner R.B., Wang F., Berkstresser B., Kim J., Wang T.J., Lewis G.D., Hutter A.M., Picard M.H., Baggish A.L. (2012). Regression of “gray zone” exercise-induced concentric left ventricular hypertrophy during prescribed detraining. J. Am. Coll. Cardiol..

[16] Evangelista F.S., Martuchi S.E., Negrão C.E., Brum P.C. (2005). Loss of resting bradycardia with detraining is associated with intrinsic heart rate changes. Braz. J. Med. Biol. Res..

[17] Bánhegyi A., Pavlik G., Olexó Z. (1999). The effect of detraining on echocardiographic parameters due to injury. Acta Physiol. Hung..

[18] Chen Y.T., Hsieh Y.Y., Ho J.Y., Lin T.Y., Lin J.C. (2022). Two weeks of detraining reduces cardiopulmonary function and muscular fitness in endurance athletes. Eur. J. Sport Sci..

[19] Coyle E.F., Hemmert M.K., Coggan A.R. (1986). Effects of detraining on cardiovascular responses to exercise: Role of blood volume. J. Appl. Physiol.

[20] Ready A.E., Quinney H.A. (1982). Alterations in anaerobic threshold as the result of endurance training and detraining. Med. Sci. Sports Exerc..

[21] Coyle E.F., Martin W.H., Sinacore D.R., Joyner M.J., Hagberg J.M., Holloszy J.O. (1984). Time course of loss of adaptations after stopping prolonged intense endurance training. J. Appl. Physiol. Respir. Environ. Exerc. Physiol..

[22] Zheng J., Pan T., Jiang Y., Shen Y. (2022). Effects of short- and long-term detraining on maximal oxygen uptake in athletes: A systematic review and meta-analysis. Biomed. Res. Int..

[23] Hickson R.C., Rosenkoetter M.A. (1981). Reduced training frequencies and maintenance of increased aerobic power. Med. Sci. Sports Exerc..

[24] Spiering B.A., Mujika I., Sharp M.A., Foulis S.A. (2021). Maintaining physical performance: The minimal dose of exercise needed to preserve endurance and strength over time. J. Strength Cond. Res..

[25] Lemmer J.T., Hurlbut D.E., Martel G.F., Tracy B.L., Ivey F.M., Metter E.J., Fozard J.L., Fleg J.L., Hurley B.F. (2000). Age and gender responses to strength training and detraining. Med. Sci. Sports Exerc..

[26] Taaffe D.R., Marcus R. (1997). Dynamic muscle strength alterations to detraining and retraining in elderly men. Clin. Physiol..

[27] Correa C.S., Baroni B.M., Radaelli R., Lanferdini F.J., Cunha Gdos S., Reischak-Oliveira Á., Vaz M.A., Pinto R.S. (2013). Effects of strength training and detraining on knee extensor strength, muscle volume and muscle quality in elderly women. Age.

[28] Staron R.S., Leonardi M.J., Karapondo D.L., Malicky E.S., Falkel J.E., Hagerman F.C., Hikida R.S. (1991). Strength and skeletal muscle adaptations in heavy-resistance-trained women after detraining and retraining. J. Appl. Physiol..

[29] Hortobágyi T., Houmard J.A., Stevenson J.R., Fraser D.D., Johns R.A., Israel R.G. (1993). The effects of detraining on power athletes. Med. Sci. Sports Exerc..

[30] García-Pallarés J., Carrasco L., Díaz A., Sánchez-Medina L. (2009). Post-season detraining effects on physiological and performance parameters in top-level kayakers: Comparison of two recovery strategies. J. Sports Sci. Med..

[31] Vuori I., Heinonen A., Sievänen H., Kannus P., Pasanen M., Oja P. (1994). Effects of unilateral strength training and detraining on bone mineral density and content in young women: A study of mechanical loading and deloading on human bones. Calcif. Tissue Int..

[32] Delshad M., Ghanbarian A., Mehrabi Y., Sarvghadi F., Ebrahim K. (2013). Effect of strength training and short-term detraining on muscle mass in women aged over 50 years old. Int. J. Prev. Med..

[33] Padilha C.S., Ribeiro A.S., Fleck S.J., Nascimento M.A., Pina F.L., Okino A.M., Venturini D., Barbosa D.S., Mayhew J.L., Cyrino E.S. (2015). Effect of resistance training with different frequencies and detraining on muscular strength and oxidative stress biomarkers in older women. Age.

[34] Blocquiaux S., Gorski T., Van Roie E., Ramaekers M., Van Thienen R., Nielens H., Delecluse C., De Bock K., Thomis M. (2020). The effect of resistance training, detraining and retraining on muscle strength and power, myofibre size, satellite cells and myonuclei in older men. Exp. Gerontol..

[35] Borland M., Bergfeldt L., Cider Å., Rosenkvist A., Jakobsson M., Olsson K., Lundwall A., Andersson L., Nordeman L. (2022). Effects of 3 months of detraining following cardiac rehabilitation in patients with atrial fibrillation. Eur. Rev. Aging Phys. Act..

[36] Tofas T., Fatouros I.G., Draganidis D., Deli C.K., Chatzinikolaou A., Tziortzis C., Panayiotou G., Koutedakis Y., Jamurtas A.Z. (2021). Effects of cardiovascular, resistance and combined exercise training on cardiovascular, performance and blood redox parameters in coronary artery disease patients: An 8-Month training-detraining randomized intervention. Antioxidants.

[37] Aune D., Schlesinger S., Leitzmann M.F., Tonstad S., Norat T., Riboli E., Vatten L.J. (2021). Physical activity and the risk of heart failure: A systematic review and dose-response meta-analysis of prospective studies. Eur. J. Epidemiol..

[38] Hadžović-Džuvo A., Valjevac A., Lepara O., Pjanić S., Hadžimuratović A., Mekić A. (2014). Oxidative stress status in elite athletes engaged in different sport disciplines. Bosn. J. Basic Med. Sci..

[39] Ávila-Gandía V., Ramos-Campo D.J., García-Sánchez E., Luque-Rubia A.J., López A., López-Román F.J. (2023). Training, detraining and retraining effects of moderate vs. high intensity exercise training programme on cardiovascular risk factors. J. Hypertens..

[40] Merghani A., Malhotra A., Sharma S. (2016). The U-shaped relationship between exercise and cardiac morbidity. Trends Cardiovasc. Med..

[41] Pelliccia A., Lemme E., Maestrini V., Di Paolo F.M., Pisicchio C., Di Gioia G., Caselli S. (2018). Does sport participation worsen the clinical course of hypertrophic cardiomyopathy? Clinicaloutcome of hypertrophiccardiomyopathy in athletes. Circulation.

[42] Pelliccia A., Borrazzo C., Caselli S., Lemme E., Musumeci M.B., Maestrini V., Francia P., Russo D., Pelliccia M., Olivotto I. (2022). Neither athletic training nor detraining affects LV hypertrophy in adult, low-risk patients with HCM. JACC Cardiovasc. Imaging.

[43] Finocchiaro G., Papadakis M., Robertus J.L., Dhutia H., Steriotis A.K., Tome M., Mellor G., Merghani A., Malhotra A., Behr E. (2016). Etiology of Sudden death in sports: Insights from a United Kingdom Regional Registry. J. Am. Coll. Cardiol..

[44] James C.A., Bhonsale A., Tichnell C., Murray B., Russell S.D., Tandri H., Tedford R.J., Judge D.P., Calkins H. (2013). Exercise increases age-related penetrance and arrhythmic risk in arrhythmogenic right ventricular dysplasia/cardiomyopathy-associated desmosomal mutation carriers. J. Am. Coll. Cardiol..

[45] Biffi A., Palermi S., D’Ascenzi F., Bonifazi M., Zorzi A., Corrado D. (2024). Premature ventricular beats in athletes: To detrain or not to detrain?. Br. J. Sports Med..

[46] Biffi A., Maron B.J., Verdile L., Fernando F., Spataro A., Marcello G., Ciardo R., Ammirati F., Colivicchi F., Pelliccia A. (2004). Impact of physical deconditioning on ventricular tachyarrhythmias in trained athletes. J. Am. Coll. Cardiol..

[47] Gasperetti A., Dello Russo A., Busana M., Dessanai M., Pizzamiglio F., Saguner A.M., TeRiele A.S.J.M., Sommariva E., Vettor G., Bosman L. (2020). Novel risk calculator performance in athletes with arrhythmogenic right ventricular cardiomyopathy. Heart Rhythm.

[48] Wang W., Orgeron G., Tichnell C., Murray B., Crosson J., Monfredi O., Cadrin-Tourigny J., Tandri H., Calkins H., James C.A. (2018). Impact of exercise restriction on arrhythmic risk among patients with arrhythmogenic right ventricular cardiomyopathy. J. Am. Heart Assoc..

[49] de Gregorio C., Speranza G., Magliarditi A., Pugliatti P., Andò G., Coglitore S. (2012). Detraining-related changes in left ventricular wall thickness and longitudinal strain in a young athlete likely to have hypertrophic cardiomyopathy. J. Sports Sci. Med..

[50] Di Gioia G., Maestrini V., Colella A., Mango R., Segreti A., Squeo M.R., Lemme E., Pelliccia A. (2023). Reversible apical hypertrophy in a young competitive athlete with familiar hypertrophic cardiomyopathy. Am. J. Case Rep..

[51] Vessella T., Cardillo R., Bianco M., Palmieri V., Zeppilli P. (2013). Marked negative T waves in athletes: ECG normalization after detraining. J. Sports Med. Phys. Fit..

[52] Ghani S., Sharma S. (2012). Electrocardiographic changes in an athlete before and after detraining. BMJ Case Rep..

[53] Klempfner R., Kamerman T., Schwammenthal E., Nahshon A., Hay I., Goldenberg I., Dov F., Arad M. (2015). Efficacy of exercise training in symptomatic patients with hypertrophic cardiomyopathy: Results of a structured exercise training program in a cardiac rehabilitation center. Eur. J. Prev. Cardiol..

[54] Lin M.T., Chen W.C., Wu C.H., Chen S.Y., Lee C.M. (2020). The effectiveness of cardiac rehabilitation in non-ischemic dilated cardiomyopathy patients: A pilot study. J. Formos. Med. Assoc..

[55] Joo C.H. (2018). The effects of short term detraining and retraining on physical fitness in elite soccer players. PLoS ONE.

